# Retropectoral hematoma: A rare complication following biventricular implantable cardiac defibrillator upgrade

**DOI:** 10.1016/j.hrcr.2021.01.016

**Published:** 2021-02-03

**Authors:** Michael Soos, Minhazur Rahman, Ian Laxina, Daniel Gonzalez-Morales, Divyakant Gandhi, Awais Kang, Khalil Kanjwal

**Affiliations:** ∗ McLaren Greater Lansing Hospital, Lansing, Michigan; † Department of Medicine, Michigan State University, East Lansing, Michigan

**Keywords:** Defibrillator, Hematoma, Pocket

## Introduction

Cardiac implantable electronic device (CIED) implantation is a common electrophysiology procedure and almost 300,000 devices are implanted yearly.^1^ The procedure of CIED implantation is safe, with the risk of complication close to 1%. Complications may include infections, bleeding and pocket hematoma, lead dislodgement, perforation, pericardial effusion, and tamponade. The most common reported complication is pocket hematoma.

Bleeding in the pocket can lead to significant discomfort, increases risk of infection, and may require surgical drainage. While hematoma formation has not been associated with increased mortality, it can lead to increased length of stay and costs of hospitalizations.^2^ There are multiple sources from which bleeding in the pocket may occur and result in hematoma formation. These include injury to superficial veins, vascular breast tissue, insertion site vessel, and muscular arterial branches. The bleeding from these sites usually results in the bleeding in the pocket. In this report we present a rare location of bleeding into the subpectoral muscle after upgrade of a dual-chamber pacemaker to biventricular implantable cardiac defibrillator (BIV-ICD).

**Key Teaching Points**

• Hematoma is a common complication following cardiac device implantation.
• Usually, hematoma results in bleeding within the pocket.
• Physicians should be aware of the rare occurrence of the subpectoral hematoma, which may need incision of the pectoral muscle for evacuation.

## Case report

A 72-year-old woman with a history of drug-refractory permanent atrial fibrillation, prior atrioventricular node ablation, dual-chamber pacemaker insertion, and persistent cardiomyopathy with ejection fraction 20% and NYHA class III and episodes of nonsustained ventricular tachycardia noted on device telemetry was referred to our clinic for upgrade to BIV-ICD. She was on apixaban, which was continued perioperatively. She underwent successful upgrade of the dual-chamber pacemaker to BIV-ICD from a right subclavian approach. During the procedure there was extensive fibrosis noted in the floor of the pocket of the previous device. The pocket was expanded using blunt dissection and plasma blade. The scalpel was not used for pocket expansion.

The axillary subclavian venous system was patent. There was no difficulty in obtaining venous access with a micropuncture needle and hence the venous cutdown was not performed.

However, there was difficulty noted while advancement of venous sheaths and serial predilation was performed with various dilators before 2 9F sheaths could be advanced in the subclavian vein. The high-voltage lead and a coronary sinus lead were implanted. The lead parameters were acceptable. There was no evidence of bleeding noted in the pocket and it was closed in the subcutaneous layer. The patient had a normal blood pressure in the range of 95/65 mm Hg to 110/70 mm Hg throughout the procedure.

**Figure 1:**
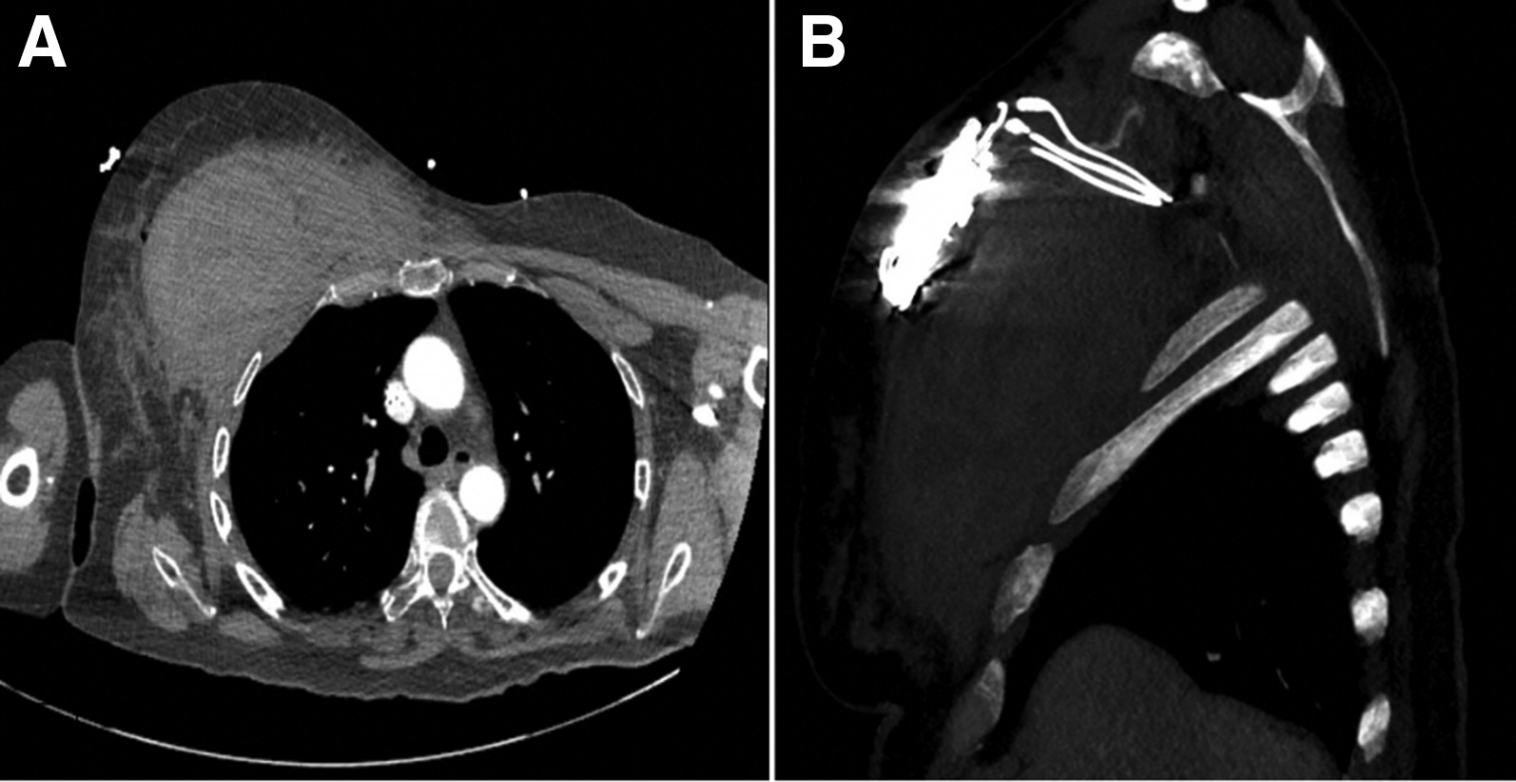
Computed tomography of the chest revealing large right subpectoral hematoma. **A:** Transverse view. **B:** Sagittal view.

**Figure 2:**
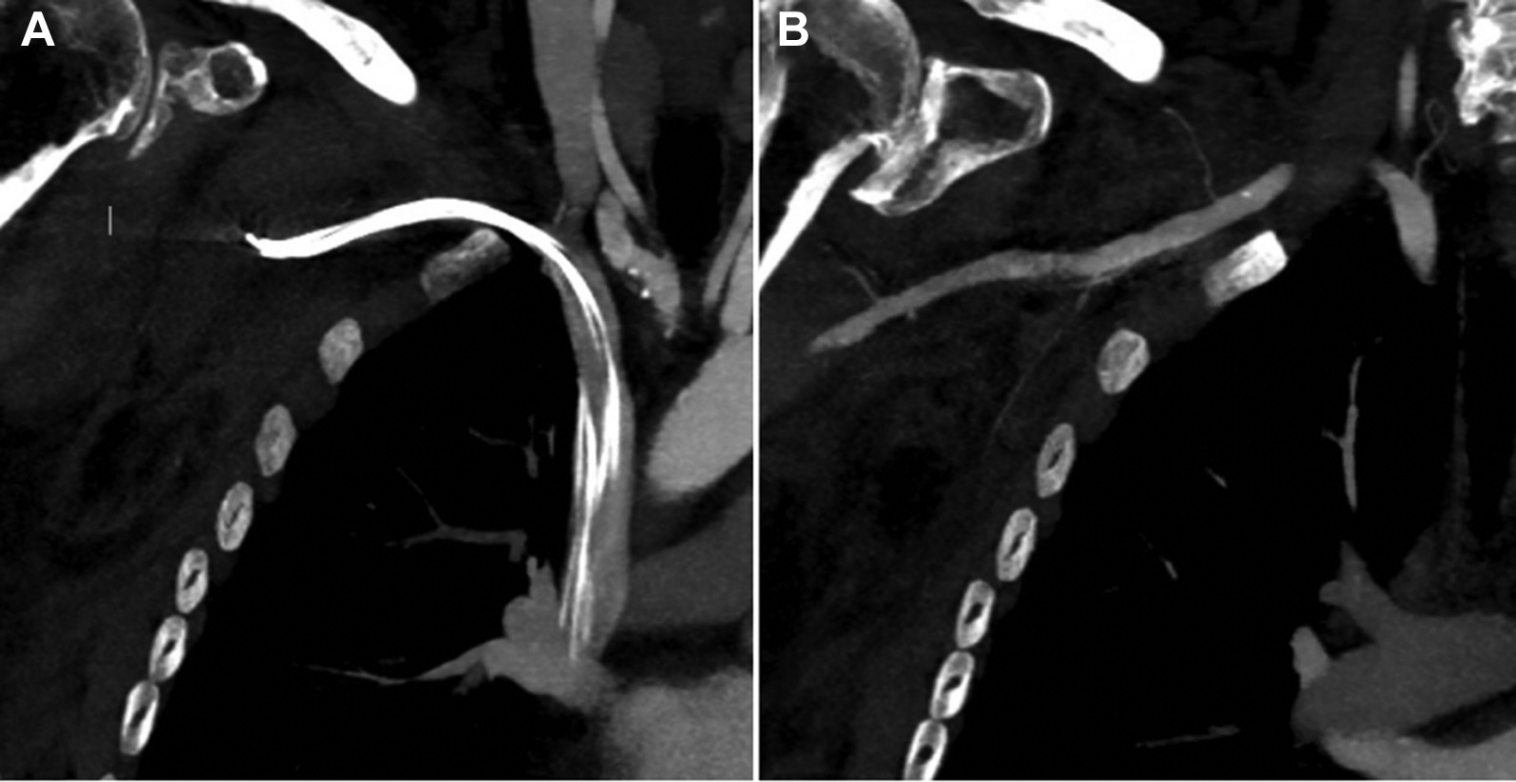
Computed tomography scan of the chest in the coronal view revealing (**A**) biventricular implantable cardiac defibrillator leads entering the axillary/subclavian vein and (**B**) active bleeding from the pectoral branch of the thoracoacromial artery.

Unfortunately, a few hours after the procedure the patient developed hematoma with a tense swelling at the procedure site. She started having pain and nausea and subsequently became hypotensive. The patient became tachypneic (respiratory rate 24 breaths/min), tachycardic (heart rate 100 beats/min), and hypotensive (blood pressure 70/45 mm Hg), After initial resuscitation an urgent computed tomography (CT) angiography of the thorax was suggestive of a large hematoma of the right pectoralis and subpectoralis musculature (Figure 1). The hematoma measured 8 × 12 × 11 cm. It also showed evidence of active extravasation at the superior margin of the hematoma, likely from a muscular branch vessel (Figure 2). Given the hemodynamic instability and evidence of active extravasation on CT imaging, the patient was taken back to the electrophysiology lab. An incision was made in the previous pocket. The lead and the devices were taken out of the pocket. When the pocket was opened there was no bleeding noted in the pocket. However, there was tense and bulging swelling noted in the subpectoral area. An incision was made with the help of an electrocautery in the longitudinal direction and parallel to the muscle fibers and almost 200 cc of blood and clots were evacuated. After complete evacuation of blood from the subpectoral area the pectoral muscle was sutured with an absorbable vicryl. The pocket was inspected again and there were no signs of active bleeding noted. The device and the leads were inserted back into the pocket, and the device was anchored to the underlying subpectoral muscle, followed by closure of the pocket in the subcutaneous layers. The patient recovered well afterwards and was discharged home in stable condition.

## Discussion

Age; history of congestive heart failure, coagulopathy, and renal failure; and clopidogrel are known risk factors for hematoma formation following CIED implantation. Clopidogrel therapy significantly increased incidence of pocket hematoma (18.3% on therapy, 10.5% when recently discontinued, and 7.9% off of therapy) and patients receiving ICDs more frequently developed hematoma.^3^ Uninterrupted warfarin use was associated with a significant reduction in pocket hematoma formation when compared to heparin bridge.^4^ However, in a similarly designed study the uninterrupted use of novel anticoagulants was not associated with reduction in pocket hematoma formation.^5^

To the best of our knowledge, subpectoral hematoma following device implantation has not been reported previously. The mechanism of subpectoral hematoma reported in various case studies includes direct blunt trauma to the chest wall and following closure of sternotomy incision during open heart surgery.^6^^,^^7^ In both cases the source of bleeding was discovered to be the internal mammary artery. The right internal mammary artery (RIMA), also known as the right internal thoracic artery, originates directly from the proximal portion of the subclavian artery. The RIMA courses inferomedially, entering the thoracic cavity beneath the first rib and clavicle before terminating distally when dividing into the superior epigastric and musculophrenic arteries. Clinically, bleeding from RIMA presents as rapidly expanding chest wall hematomas over the course of several hours associated with severe pain, nausea, and occasional vomiting.

It is possible that inadvertent trauma to the branch of the RIMA during predilation of veins could have occurred and led to submuscular bleeding. However, it is quite possible that the bleeding source was an intramuscular branch. Usually, trauma to the muscular branch is seen with bleeding in the pocket of the implanted device. In our case there was a fibrous capsule in the floor of the pocket, which could have allowed for seepage of the blood in the muscle. This could have been enhanced with the continued anticoagulation use in the perioperative period.

Given the number of years elapsed since the time of initial implantation, a number of factors related to pocket formation may have contributed to the subpectoral hematoma. In general, early wound healing after the superficial incision and subsequent plane dissection for pocket formation involves a process of “inflammation, re-epithelialization, keratinocyte proliferation, matrix metalloproteinase deposition, angiogenesis, and ultimately wound contraction and closure.”^8^ Furthermore, the immune response to a retained foreign body (CIED) incorporates a complex cascade of events involving the expression of transforming growth factor-B, which ultimately culminate in capsular formation.^8^ Histopathology of these capsules has demonstrated most commonly a composition of fibrous, fibroconnective, and fibroadipose tissue, with varying levels of acute and/or chronic inflammation, fibrosis, giant cell or granulomatous foreign body reaction, hyalinization, and calcification.^9^ Electron microscopy reveals significant contractile myofibroblastic proliferation in the pacemaker capsule.^10^ Thus, there is definitive evidence of pathophysiological fibrotic response following CIED implantation. In our particular case, we surmise that the formation of dense fibrotic capsule that encases the device pocket had in fact insulated the pocket itself from hemorrhage at an adjacent site, explaining why we could not see any bleeding when the pocket was opened. Thus, while the hematoma had clearly developed and was clinically apparent on gross examination, the subsequent direct pocket investigation did not reveal any vestige of hemorrhagic content. We believe that the hematoma formation had a tamponade effect on the bleeding vessel and stopped further bleeding, thus explaining why there was no active bleeding noted during exploration of the subpectoral area. Given the extension of the hematoma towards the axilla on CT imaging, the hematoma appears to have been encased posteriorly by the clavipectoral fascia, inferiorly by the axillary fascia, anteriorly by the pectoralis major, and superiorly by the clavicle. The bleeding arterial branch must have bled into this limited potential space; however, it was ultimately compressed posteriorly by the hematoma, circumferentially by the pectoralis major muscle, and anteriorly by both the pectoralis fascia and the overlying fibrotic capsule. We therefore believe that the additive effect of the aforementioned sequelae is responsible for both the absence of in-pocket hemorrhagic content and the obscurity of an actively bleeding vessel.

Also, it is important to note that our patient was on uninterrupted apixaban in the perioperative period. There was no increased risk of bleeding noted in patients with continued novel anticoagulants in the perioperative period of device implant.^5^ We do not suggest any change in the practice of perioperative use of novel anticoagulation based on our single case report. However, we believe that patients with continued anticoagulation are likely to bleed more if a complication like a vascular injury happens during the procedure. We also believe the patients undergoing generator change-outs and upgrades with fibrotic capsules and continued anticoagulation are likely to bleed more in the pocket. Physicians need to have a high suspicion of retropectoral hematoma in this patient population. In these high-risk patients, it may also be helpful to apply firm manual pressure for 5–10 minutes on the pocket to help prevent bleeding from any occult subpectoral source.

## Conclusion

Intramuscular bleeding can be a rare complication following CIED implantation and can lead to re-exploration of the pocket. Physicians need to be aware of this complication.
